# A new 3D printing porous trabecular titanium metal acetabular cup for primary total hip arthroplasty: a minimum 2-year follow-up of 92 consecutive patients

**DOI:** 10.1186/s13018-020-01913-1

**Published:** 2020-09-04

**Authors:** Xiao Geng, Yang Li, Feng Li, Xinguang Wang, Ke Zhang, Zhongjun Liu, Hua Tian

**Affiliations:** Department of Orthopaedics, Peking University Third Hospital, No. 49 North Garden Road, Beijing, 100191 China

**Keywords:** Arthroplasty, Hip, 3D printing, Trabecular titanium acetabular cup, Outcome

## Abstract

**Background:**

Aseptic cup loosening is still one of the main reasons leading to acetabular cup failures. 3D printing porous trabecular titanium metal acetabular cup may provide good initial stability and secondary fixation because of its highly interconnected, porous structure. Few large sample studies have reported the clinical outcomes of electron beam melting (EBM) porous titanium acetabular cup in Chinese population.

**Methods:**

We retrospectively collected and analyzed the clinical data of a total of 92 consecutive patients between January 2013 and November 2017, with an average follow-up of 48.2 ± 3.6 months. Clinical outcomes included Harris Hip Score (HHS), the Western Ontario and McMaster Universities (WOMAC) Osteoarthritis Index, satisfaction rate, and cup survival rate were evaluated. Radiographic assessments were conducted to evaluate osteointegration.

**Results:**

HHS scores improved significantly while the WOMAC score decreased significantly at the latest follow-up (*p* < 0.001). The satisfaction rate (prevalence of satisfied or very satisfied) was 91.3%. No acetabular cup failures occurred. Radiolucent lines appeared in 15 cases (18 hips) and disappeared in 6 months. No cup loosening signs found until the last follow-up. The overall survival rate of implantation is 99.1% (cup survival rate 100%).

**Conclusion:**

The new EBM-produced 3D ACT™ cup demonstrated us its favorable short- to mid-term clinical outcomes in Chinese THA patients. It can provide high acetabular cup survival rate, great clinical improvements and excellent biological fixation. Further investigations are needed to confirm its long-term outcomes.

## Background

Cementless acetabular cups have been utilized more and more common in total hip arthroplasty (THA) in recent years [1, 2]. Although it has provided remarkably successful clinical outcomes in primary THAs, every year many cup failures, especially aseptic cup loosening, occur and lead to intractable revisions and heavy medical burdens [3]. Thus, surgeons and engineers have been working on prosthetic designs and materials for many years to improve the acetabular cups’ primary mechanical stability and secondary bone fixation [4–6].

The mechanical fixation and bone integration between the surface of the cup and the host bone interface are important factors to maintain initial and long-term stability of the cups [7, 8]. But it is technically limited because the traditional two-step porous coated cup surface cannot be designed integrated with the solid layer and optionally to achieve a better bone osseointegration with the host bone by ideal porous structure [9].

As the 3D printing technology widely applied to design various orthopedic implants, a few studies have showed excellent clinical and radiological outcomes and survivorship of the porous trabecular titanium metal acetabular component via EBM, which is an important branch of 3D printing technology, especially when patients with poor bone quality in primary and revision THA surgeries [10, 11]. With an average porosity of 65% and a mean pore diameter of 640 μm, a kind of 3D printing porous titanium acetabular component, DELTA-TT™ cup, was reported to obtain satisfactory mid-term clinical and radiographic outcomes [12]. The continuity between the porous surface and solid parts broke through the limitations of the traditional porous coatings, which can provide a higher resistance to detachment and corrosion of the two parts. Also, the EBM trabecular titanium has showed good osteoinductive and osteoconductive performances by a series of vitro and animal experiments [13–15].

**Fig. 1:**
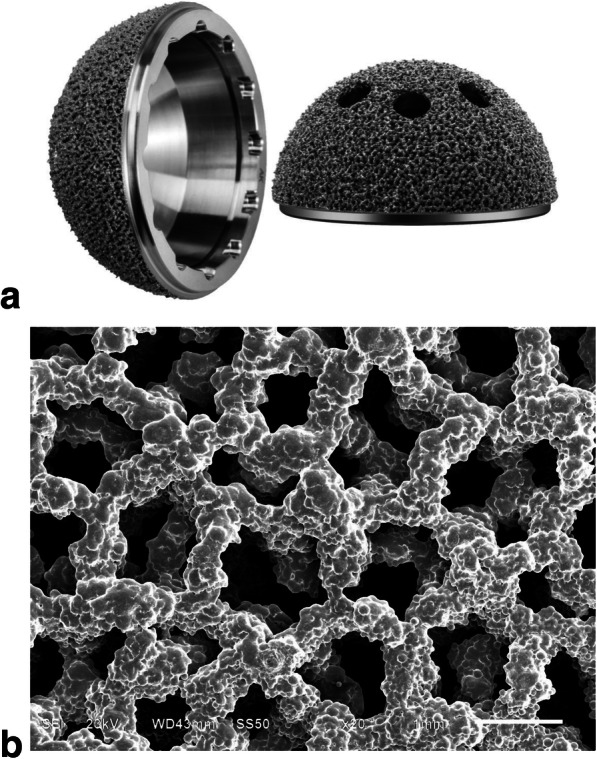
The picture shows the 3D ACT EBM-produced trabecular titanium acetabular cup (**a**) and the SEM image of its interconnected trabecular titanium cellular solid structure showed the porous architecture was designed based on a dodecahedron unit cell (**b**)

The 3D ACT™ cups (Fig. 1) were started utilized in primary THAs and hip revisions since year 2013 and performed good clinical outcomes refer to our clinical practice. Few large sample studies have reported the clinical outcomes of this kind of EBM porous titanium acetabular cup. The aim of this study was to investigate the short- to mid-term clinical and radiographic outcomes and satisfaction of 92 consecutive patients who underwent primary THA using a new 3D printing produced trabecular titanium cups, relatively for complex THA cases.

## Methods

### General data

After the approval of the Institutional Review Board was obtained for this study, we retrospectively collected the data from a total of 103 patients (120 hips) who underwent primary total hip arthroplasty used the 3D ACT porous titanium (Ti6Al4V) trabecular acetabular cups between January 2013 and November 2017. Inclusion criteria: males and females > 18 years old, with a body mass index (BMI) ≤ 40 kg/m^2^, diagnosed with primary hip osteoarthritis (OA), fracture of femoral neck (FFN), or secondary hip osteoarthritis due to developmental dysplasia of the hip (DDH), hip avascular necrosis (AVN), rheumatoid arthritis (RA), ankylosing spondylitis (AS), and coxa plana with a minimum 2-year clinical follow-up. Exclusion criteria: severe vascular diseases of the lower extremity, neuromuscular diseases, infectious diseases, severe dysfunctions of major organs, and severe neurosensory deficiencies caused by spinal diseases.

Three patients died of cancers and 8 patients were lost to follow-up before the 2-year follow-up. None of these 11 patients were deceased due to THA associated diseases or underwent revision until our last evaluation. Finally, 40 males (47 hips) and 52 females (61 hips) were enrolled in our study, with 2–6-year follow-up. Patient demographics and preoperative clinical data were listed in Table 1. Seventy-six unilateral procedures (40 left-sided and 36 right-sided) were performed and 14 patients underwent a bilateral two-stage procedure, while the 2 remaining patients underwent a simultaneous bilateral THA surgery. Original diagnosis included OA in 8 (7.4%) cases, DDH in 52 (48.1 %) cases, RA in 3(2.7%) cases, AVN in 36 (33.3%) cases, FFN in 4 (3.7 %) cases, AS in 4 (3.7 %) cases, and coxa plana in 1 (0.9%) cases.

**Table 1:** Demographic and clinical data of the patients

		Mean	SD	*p*
Age (years)	Male (*n* = 40)	59.8	9.8	0.58
	Female (*n* = 52)	58.7	8.9	
BMI (kg/m^2^)	Male (*n* = 40)	26.0	4.9	0.51
	Female (*n* = 52)	26.8	6.4	
Pre-Harris hip score		45.2	4.8	
Pre-WOMAC score		54.4	7.3	

### Components and surgical procedures

**Fig. 2:**
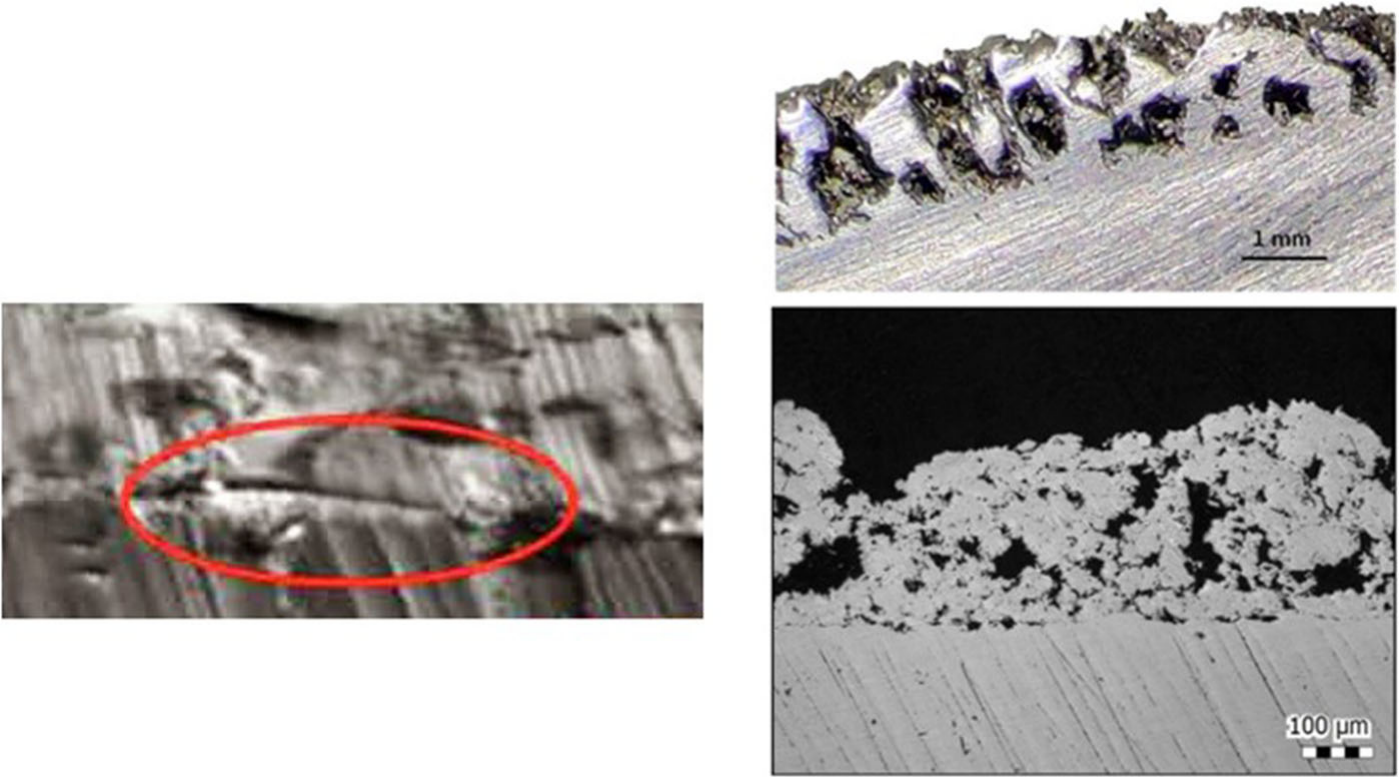
The picture shows the interface between the two layers of traditional cup (left) and the integration EBM porous structure (right). The EBM technique achieved the melting of thin layers of metal powder, modeling a bulk construct which respects the original metal alloy properties and integrates as a whole trabecular surface

Cementless 3D ACT cups (Fig. 1), ML stems, BIOLOX® Delta ceramic (CeramTec GmbH, Plochingen, Germany) femoral heads and ceramic-on-polyethylene bearing surfaces were used in all cases. The structure of 3D ACT cup is characterized by a 1.5-mm-thick porous layer, an average porosity of 80%, pore size of 600–800 μm, and lower modulus of elasticity which is equal to that of human cancellous bone. The rough surface, with coefficient of friction on cancellous bone of 1.08 and on cortical bone of 0.93, could theoretically obtain a better bone-implant initial fixation. There are 16 specifications of the 3D ACT cups, and the diameter of which is 40–70 mm, with 2 mm interval. Unlike the two-step coated surface of the traditional cup, the ACT cup was naturally integrated (Fig. 2), reducing the detachment risk of the metal solid layer and the surface.

Fifty-six posterolateral approaches and 52 modified Hardinge approaches were performed by 6 senior surgeons due to their preferences. All cups were implanted by 1 mm press-fit technology according to the manufacturers’ recommendations with additional screw fixation if necessary. Bedside pelvic X-rays to confirm implant locations and primary stabilities were performed immediately after patients were sent back to ward. Early function rehabilitation was carried out routinely. Patients were asked to walk around with walking aids the day after surgery if bedside pelvic X-rays showed no dislocations and instabilities. Analgesia protocol was in accordance with multimodal analgesia suggested by clinical guidelines. We applied intravenous Parecoxib and oral oxycodone for patients underwent THA in this study. Rivaroxaban (10 mg/day) to prevent thromboembolism and lower extremity venous ultrasound was performed on the fifth day after surgery to identify potential deep venous thrombosis (DVT) events.

**Fig. 3:**
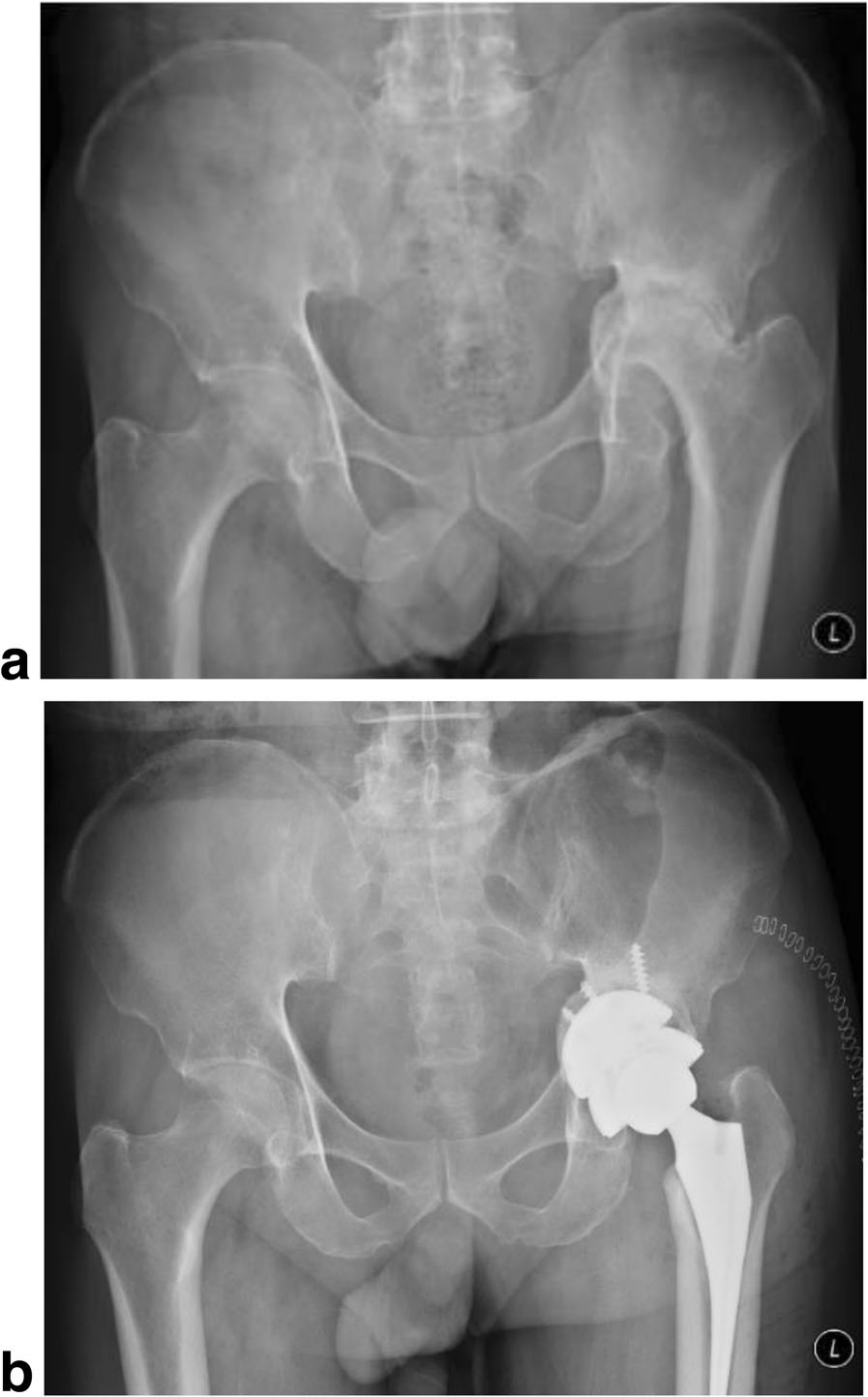
The picture shows a 64-year-old male patient with acetabular protrusion caused by severe RA (**a**), underwent a 3D ACT primary THA with 3D printing augments for bone defect repair (**b**)

No intraoperative complications occurred, although there were several considerable tough cases, like Crowe type IV DDH and RA stiffness hip combined with severe osteoporosis. In one case, a patient with acetabular protrusion secondary to severe RA, two half-moon-shaped 3D printing augments (50 mm × 15 mm, Fig. 3), designed by the same EBM technology, were used to repair bone defect before the implantation of the cup. All cups obtained good primary stabilities after implantation.

### Clinical and radiographic assessments

Patients’ clinical and radiographic data, as well as complications, were collected preoperatively and 3, 6, 12 months, then annually after 1 year postoperatively. HHS [16] and WOMAC score [17] are evaluated as clinical outcomes. A self-assessment satisfaction questionnaire (sorted by very dissatisfied, dissatisfied, neutral, satisfied, or very satisfied) was used to assess patients’ subjective satisfaction rate [18]. Postoperative complications were recorded as well.

An independent clinical staff who was blinded to each patient’s clinical condition assessed the radiographic results, including radiolucent lines, osteolysis, and sclerosis according to the DeLee and Charnley’s [19] definition of three zones and the bone ingrowth criteria defined by the Anderson Orthopedic Research Institute [20] to evaluate bone osseointegration and acetabular component stability. Cup loosening criteria: a progressive radiolucent line wider than 2 mm or cup migration more than 5 mm or cup inclination change more than 5°. Cup osseointegration criteria: (1) the absence of radiolucent lines; (2) the presence of superolateral buttresses; (3) the presence of medial stress-shielding; (4) the presence of radial trabeculae; and (5) the presence of inferomedial buttresses. No less than three signs can be determined as radiographic osseointegration. The observer was asked to perform radiographic analysis at least twice for each X-ray to confirm the judgements.

### Statistical assessments

SPSS (Version 20, IBM Corp.) was used for the statistical analysis. All continuous variables were expressed as mean and standard deviation and compared by *t* tests if they are normally distributed. An appropriate chi-square test or Fisher’s exact test was carried out to compare categorical variables. Statistical significance level was defined at *p* value < 0.05.

## Results

A total of 92 consecutive patients (108 hips) were enrolled in statistical analysis. Seventy-five (81.5%) patients completed the clinical and radiographic evaluation by regular follow-up, and data of the remaining 17(18.5%) patients were collected via telephones and/or online communications.

The mean follow-up period was 48.2 ± 3.6 months. Forty (43.5%) were males and 52 (56.5%) were females, with a mean age of 59.8 ± 9.8 years old. Average body mass index (BMI) was 26.0 ± 4.9 kg/m^2^. HHS improved remarkably, from 45.2 ± 4.8 preoperatively to 95.8 ± 6.0 postoperatively, along with WOMAC score decreased significantly from 54.4 ± 7.3 preoperatively to 11.2 ± 4.2 at the last follow-up (Table 2). The satisfaction rate (prevalence of satisfied or very satisfied) was 91.3% (84 patients).

**Table 2:** Follow-up of the clinical outcomes of patients using ACT cups (mean ± SD)

	Pre-op*	6 months post-op**	1 year post-op	2 years post-op	Last follow up
Harris Hip Score	54.4 ± 7.3	16.3 ± 6.2†	13.6 ± 5.8†	10.5 ± 3.7†	11.2 ± 4.2†
WOMAC Score	45.2 ± 4.8	90.5 ± 5.3†	95.1 ± 4.5†	96.7 ± 5.2†	95.8 ± 6.0†

†*p* < 0.001 when compared with preoperative scores

*Pre-op preoperative

**Post-op postoperative

**Fig. 4:**
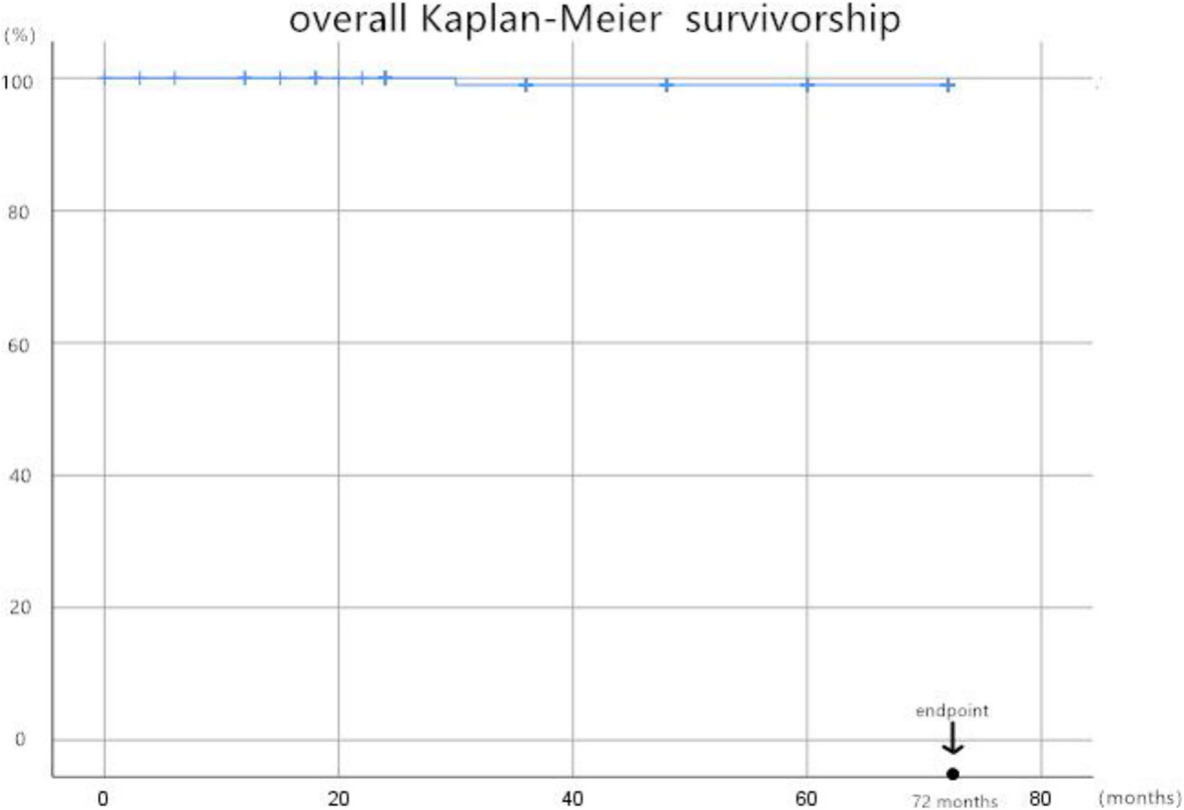
The overall Kaplan-Meier cumulative survivorship of implantation

Dislocation occurred in 1 (0.9%) female patients with Crowe IV DDH immediately after surgery, and problem was solved after an open reduction on the same day. During the first 2 years follow-up, none of the patients complained unbearable pain or dysfunction that stopped them to return to daily routine activities. One (0.9%) female patient presented to us 30 months postoperatively because of the aseptic loosening of the femoral stem, then a revision of the femoral stem-only was taken on her because we found the ACT cup fixed firmly during the surgical exploration. They were the only 2 patients who declared very dissatisfied and the other 106 (98.1%) of the patients were satisfied with the treatments. Besides, no cup revisions (cup survival rate was 100%) and other complications occurred during the follow-up period, and the overall Kaplan-Meier cumulative survivorship was 99.1% at the latest follow-up (Fig. 4).

**Fig. 5:**
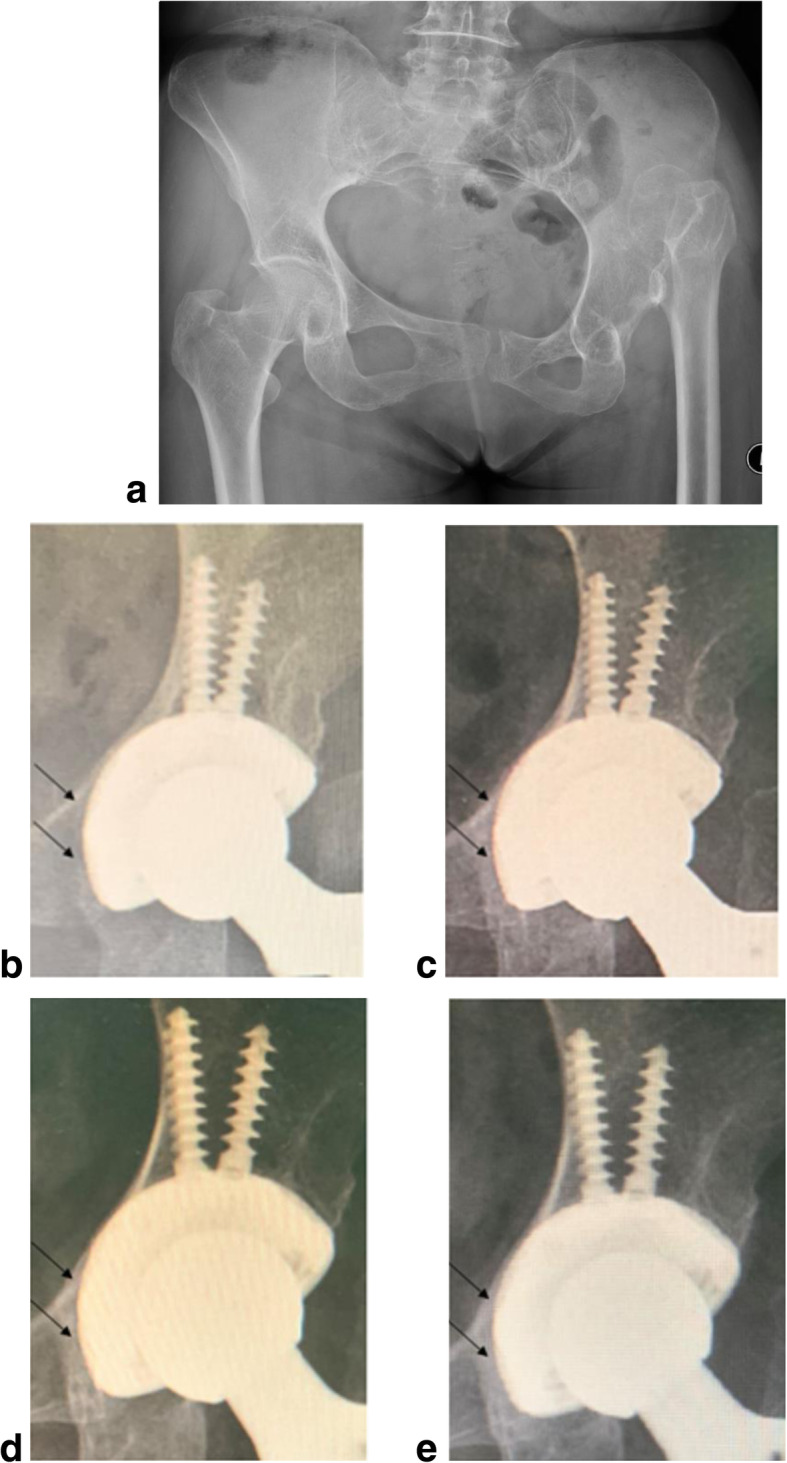
The picture shows the radiographic assessments of a 61-year-old female patient with Crowe IV DDH, preoperatively (**a**) and 2 weeks (**b**), 3 months (**c**), 6 months (**d**), and 4 years (**e**) postoperatively. Radiolucent line (appeared on 4b and 4c, black arrow) vanished at about 6 months postoperatively represents new bone ingrowth

Radiographic assessments were carried out at the latest follow-up, and all cups showed excellent osseointegration with at least three signs according to the Anderson criteria. Radiolucent lines appeared within 2 mm in 15 cases (18 hips) and disappeared in 6 months postoperatively (Fig. 5). No significant cup migration or inclination met the cup loosening criteria.

## Discussion

With the operation technology becoming more and more mature, surgeons and engineers are paying more and more attention to prosthesis designs and modifications with better biomechanics properties to improve THA clinical outcomes [21, 22]. The absence of locking mechanism of bone cement may lead to high early aseptic loosening, so cementless acetabular and femoral components have become more and more popular in THA surgeries [23]. Multiple studies report short- to mid-term clinical outcomes with a large variety of cementless acetabular cups [24–26]. As is known to all, both initial mechanical stability and long-term biological fixation are essential parts to achieve durable survivorship of acetabular cups. Thus, the biological coating technology becomes a highlight issue. Traditional coated surfaces of the acetabular cup include cobalt chromium molybdenum sintered beads, diffusion-bonded fiber metal mesh, cancellous structured titanium, and titanium-sprayed plasma demonstrated a certain osseointegration ability. However, the limitations of these techniques may occur in case of poor host bone qualities and conditions, such as osteonecrosis, osteoporosis, dysplasia, and massive bone defect.

The manufacturing process of 3D printing acetabular cup is completely different from that of traditional cup. In traditional reduction casting process, the interface between the solid layer and the coated surface of the acetabular cups may cause detachment and corrosion, resulting to cup failures. But 3D printing, via additive manufacturing process, has made it easier to individualize product design and manufacturing [27]. Thus, the 3D ACT EBM cup, with more appropriate porosity, ideal porous diameter, lower modulus of elasticity, and better coefficient of friction, may provide better solutions for various kinds of primary THAs.

It has been formerly confirmed that EBM manufactured implants, with interconnected porosity and rough surface, could provide a viable medium for bone ongrowth and ingrowth by creating a stable porous construct while also maintaining the mechanical strength in vitro and animal studies [13–15]. Perticarini [12] et al. first reported the successful utilization of trabecular titanium acetabular cups in European patients, among which are mainly primary hip OA, as well as AVN and DDH patients, and significant functional recovery and pain relief were recorded in all 133 cases at minimum 5 years follow-up.

In our study, all 3D ACT cups obtained initial stabilities and long-term fixation. As is showed in the results section, HHS improved remarkably from 45.2 ± 4.8 preoperatively to 95.8 ± 6.0 postoperatively, along with WOMAC score decreased significantly from 54.4 ± 7.3 preoperatively to 11.2 ± 4.2 at the last follow-up. Pain relief and functional recovery were as good as that reported in previous studies. High cup survival rate (99.1%) and patient satisfaction rate (91.3%) illustrated excellent surgical safety and efficiency. No cup loosening determined according to the DeLee and Charnley’s definition of three zones and excellent bone ingrowth defined by the Anderson Orthopedic Research Institute. Early-stage radiolucent lines within 2 mm showed in 15 cases gradually disappeared at 3 to 6 months postoperatively, which represented efficient bone ingrowth to the porous metal structure of this 3D ACT cup. The interface micromotion can be reduced if the coefficient of friction get increased, which can enhance primary fixation and contribute to secondary fixation [28, 29]. Great bone integration property is considered as the EBM-produced cups’ peculiar advantage related to the following: ① its extremely more rough surface and higher coefficient of friction on cancellous bone of 1.08, compared with that in traditional sintered beads (0.5); and ② its porous, solid, and special interconnected structure mimic the trabecular morphology of the natural bone. These properties do have the advantages of osteoinduction and osteoconduction by stimulate vascularization, osteoblast proliferation, and differentiation in previous study [30].

It should be mentioned that in China, the biggest developing country in the world, we often encounter severe type of end-stage hip diseases, such as Crowe IV DDH and acetabular protrusion with great bone defect, due to China’s national economical and healthcare conditions. In our study, we are glad to see encouraging great clinical and radiographic outcomes in difficult cases such as Crowe IV DDH, acetabular protrusion with large bone defect, and RA patients with severe osteoporosis. Remarkable HHS and WOAMC score improvements and high satisfaction rate ensured the cup’s efficiency among Chinese patients. No acetabular cup failures occurred in any cases, with 100% survival rate till the last follow-up, together showed an excellent short to mid-term outcomes of the 3D ACT cups.

This study does have several limitations. First, in this retrospective study, 11/103 of the patients lost to follow-up. Second, no controlled groups enrolled in this study, and we are about to carry out a prospective randomized controlled trial for higher-level evidence. Third, cases from six different surgeons enrolled which may confound the results. Fourth, computer tomography (CT) scans and bone densitometry, evaluations as well as relevant laboratory examinations were not conducted.

## Conclusions

As far as we are concerned, the application of EBM-produced 3D ACT cup demonstrated us its favorable short- to mid-term clinical outcomes in Chinese THA patients. It can provide high acetabular cup survival rate, great clinical improvements, and excellent biological fixation. More investigations of the outcomes of this EBM-produced porous trabecular titanium cup are needed in larger volume of patients and at longer term follow-up.

## Data Availability

Upon request, raw data can be provided.

## Abbreviations

EBM: Electron beam melting
HHS: Harris Hip Score
WOMAC: the Western Ontario and McMaster Universities
BMI: Body mass index
OA: Osteoarthritis
FFN: Fracture of femoral neck
DDH: Developmental dysplasia of the hip
AVN: Avascular necrosis
RA: Rheumatoid arthritis
AS: Ankylosing spondylitis
