# Correction to: A new 3D printing porous trabecular titanium metal acetabular cup for primary total hip arthroplasty: a minimum 2-year follow-up of 92 consecutive patients

**DOI:** 10.1186/s13018-020-02095-6

**Published:** 2020-11-26

**Authors:** Xiao Geng, Yang Li, Feng Li, Xinguang Wang, Ke Zhang, Zhongjun Liu, Hua Tian

**Affiliations:** Department of Orthopaedics, Peking University Third Hospital, No. 49 North, Garden Road, Beijing, 100191 China

**Correction to: J Orthop Surg Res 15, 383 (2020)**

**https://doi.org/10.1186/s13018-020-01913-1**

Following publication of the original article [1], at the request of the copyright holder of the image in the bottom right panel of Fig. 2, the authors have replaced this figure with the following figure:

**Fig. 2:**
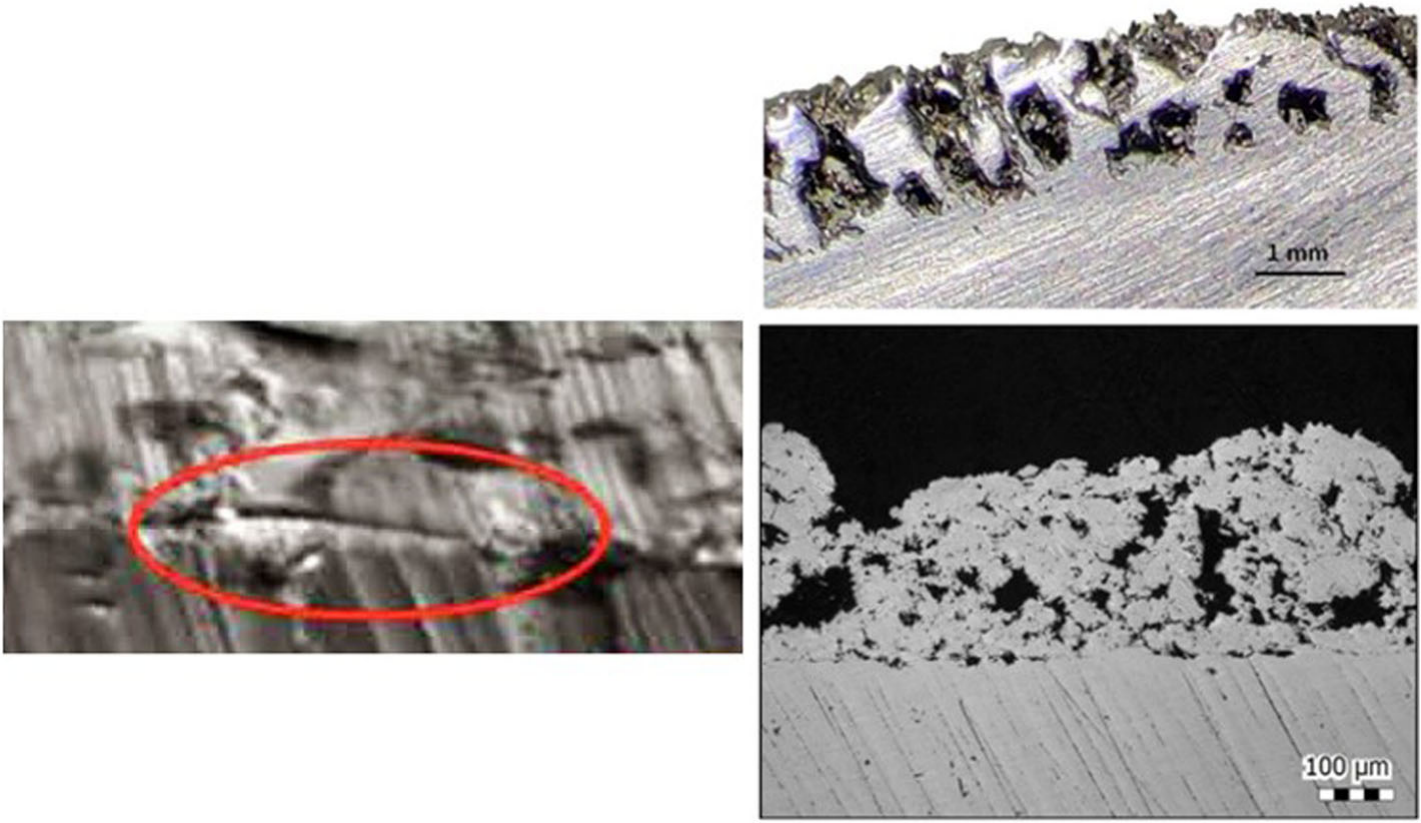
The picture shows the interface between the two layers of traditional cup (left) and the integration EBM porous structure (right). The EBM technique achieved the melting of thin layers of metal powder, modeling a bulk construct which respects the original metal alloy properties and integrates as a whole trabecular surface

The original article has been corrected.
